# Experimentally increased brood size accelerates actuarial senescence and increases subsequent reproductive effort in a wild bird population

**DOI:** 10.1111/1365-2656.13186

**Published:** 2020-03-12

**Authors:** Jelle J. Boonekamp, Christina Bauch, Simon Verhulst

**Affiliations:** ^1^ Groningen Institute for Evolutionary Life Sciences University of Groningen Groningen The Netherlands; ^2^ Centre for Ecology & Conservation School of Biosciences University of Exeter Penryn UK

**Keywords:** actuarial senescence, antagonistic pleiotropy, carry‐over effects, evolution of ageing, reproductive restraint, terminal investment, Williams

## Abstract

1. The assumption that reproductive effort decreases somatic state, accelerating ageing, is central to our understanding of life‐history variation. Maximal reproductive effort early in life is predicted to be maladaptive by accelerating ageing disproportionally, decreasing fitness.
2. Optimality theory predicts that reproductive effort is restrained early in life to balance the fitness contribution of reproduction against the survival cost induced by the reproductive effort. When adaptive, the level of reproductive restraint is predicted to be inversely linked to the remaining life expectancy, potentially resulting in a terminal effort in the last period of reproduction.
3. Experimental tests of the reproductive restraint hypothesis require manipulation of somatic state and subsequent investigation of reproductive effort and residual life span. To our knowledge the available evidence remains inconclusive, and hence reproductive restraint remains to be demonstrated.
4. We modulated somatic state through a lifelong brood size manipulation in wild jackdaws and measured its consequences for age‐dependent mortality and reproductive success.
5. The assumption that lifelong increased brood size reduced somatic state was supported: Birds rearing enlarged broods showed subsequent increased rate of actuarial senescence, resulting in reduced residual life span.
6. The treatment induced a reproductive response in later seasons: Egg volume and nestling survival were higher in subsequent seasons in the increased versus reduced broods' treatment group. We detected these increases in egg volume and nestling survival despite the expectation that in the absence of a change in reproductive effort, the reduced somatic state indicated by the increased mortality rate would result in lower reproductive output. This leads us to conclude that the higher reproductive success we observed was the result of higher reproductive effort.
7. Our findings show that reproductive effort negatively covaries with remaining life expectancy, supporting optimality theory and confirming reproductive restraint as a key factor underpinning life‐history variation.

## Graphical abstract

Early life reproductive restraint could be advantageous when it promotes health. This study compares reproductive success of jackdaws among accelerated versus decelerated ageing groups. Reproductive success increased in the accelerated ageing group, demonstrating reproductive restraint. Reproductive restraint provides an explanation for the commonly observed increase in reproductive success with age. Photo credit: Jelle Boonekamp.

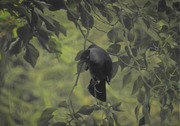

## 1 INTRODUCTION

A fundamental characteristic of life‐history diversity is that rates of reproduction and survival are negatively correlated across species (Jones et al., 2008). Such correlations may reflect a life‐history trade‐off between reproductive effort and residual reproductive value, for example via differential resource allocation between reproduction and somatic state (Kirkwood, 1977; Kirkwood & Rose, 1991). Optimality theory (Parker & Smith, 1990) predicts that selection favours parents that optimize their solution to the trade‐off between current reproductive effort versus somatic maintenance and future reproduction, maximizing fitness (McNamara & Houston, 1996). The optimality framework underpins our understanding of life‐history diversity, including ageing and life span, yet it remains elusive when and to what extent survival and reproduction trajectories causally interact.

Life‐history trade‐offs have been particularly well‐studied in wild birds. A general observation of these studies is that birds can successfully raise young added to their natural broods, raising the question of why the natural brood size appears to be lower than the parental capacity for reproductive effort (Vander Werf, 1992). One explanation for this phenomenon is that restrained reproductive effort maximizes fitness when the benefits of the increased reproductive effort to the maximum sustainable level are outweighed by the ensuing fitness costs (i.e. reduced survival and reproduction; Williams, 1966). Under this ‘reproductive restraint’ hypothesis, a reduced life expectancy is expected to induce an increase in reproductive effort. This can easily be seen for the extreme case that remaining life expectancy is zero, because in this situation there are no benefits of reproductive restraint.

Although the theory predicts reproductive restraint to be a general feature of iteroparous organisms, experimentally demonstrating this phenomenon has proven difficult. That most bird species can raise extra young (Vander Werf, 1992) can superficially be considered as evidence for reproductive restraint. However, offspring in enlarged broods generally grow to a smaller size (Dijkstra et al., 1990) suggesting that extra young are reared at the expense of their quality instead of the parent's residual reproductive value. Thus, the fact that parents can raise additional offspring does by itself not prove reproductive restraint. A further difficulty in demonstrating reproductive restraint is the confounding effects of other variables such as age and somatic state. For example, increased reproductive output prior to death can be interpreted as a terminal effort, implicitly assuming that reproductive effort was restrained in the time leading up to that point, but, alternatively, death was the consequence of the increased reproductive effort and these interpretations are difficult to separate using non‐experimental data (Clutton‐Brock, 1984; Hamel, Côté, & Bianchet, 2011; Morin, Rughetti, Rioux‐Paquette, & Festa‐Bianchet, 2016; Rughetti, Dematteis, Meneguz, & Festa‐Bianchet, 2014). Moreover, reproductive restraint may be difficult to detect because reproductive senescence with chronological age could mask the effects of reduced reproductive restraint. We consider therefore that demonstrating reproductive restraint requires an experimental approach that on the one hand induces a reduction in remaining life span, while on the other hand inducing an increase in subsequent reproductive effort relative to individuals in which remaining life span was not reduced.

We manipulated reproductive effort through brood size manipulation to manipulate somatic state, because reproductive effort is generally assumed to decrease somatic state (Gaillard & Lemaitre, 2017; Kirkwood, 1977; Williams, 1957). Brood size manipulation is a well‐established tool for the manipulation of parental reproductive effort in birds (e.g. Santos & Nakagawa, 2012). We use this approach to simultaneously estimate the fitness cost of reproductive effort in terms of its effect on residual life span, and the associated reproductive response in subsequent years, to test the reproductive restraint hypothesis. Our experimental approach allows teasing apart the effects of senescence and reproductive restraint by estimating the effect of the reproductive effort manipulation over and above relationships with chronological age.

**FIGURE 1:**
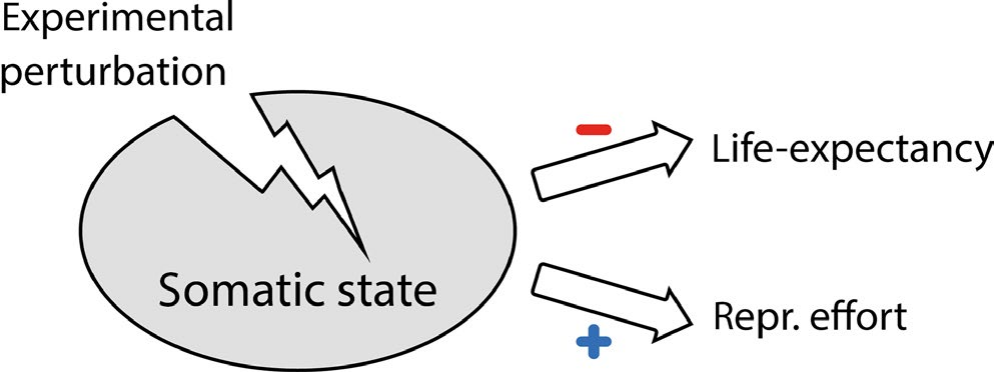
Schematic depicting the life‐history consequences of reduced somatic state by experimental perturbation as predicted by the reproductive restraint hypothesis. A perturbation reducing somatic state in one season induces a bivariate response reducing the remaining life expectancy on the one hand while simultaneously increasing reproductive effort in the subsequent season. Consequently, negative covariance between remaining life expectancy and reproductive effort is expected under the reproductive restraint hypothesis. In this study we use brood size manipulation to manipulate parental somatic state

We assume that the predicted negative association between life expectancy and reproductive effort emerges from a third state‐defining variable that is causal to this pattern, in the sense that low state causes reduced survival and, because of this effect, simultaneously induces an increase in reproductive effort (Figure 1). We here assume that any experimental non‐instantaneous life span reduction can be taken as evidence of the treatment successfully modulating somatic state, without requiring detailed knowledge of the underlying physiological variables that constitute somatic state (Figure 1).

We recently showed in a wild jackdaw population that brood size treatment in a single year did not affect parental survival, while *lifelong* experimentally increased brood size did accelerate actuarial senescence, that is the rate of increase in age‐dependent mortality (Boonekamp, Salomons, Bouwhuis, Dijkstra, & Verhulst, 2014, 2015). These findings, together with results from the meta‐analysis of single‐event brood size manipulations (Santos & Nakagawa, 2012), who found no clear pattern with respect to parental survival in a meta‐analysis, suggest that single manipulations either cause negative effects too small to be detected, or that birds are capable of buffering and recovering from short‐term increased effort (for a detailed discussion see Boonekamp et al., 2014). Notwithstanding the exact interpretation, the finding that longitudinally increased brood size‐accelerated actuarial senescence supports the assumption that reproductive effort affects somatic state (Kirkwood, 1977). However, it remains to be tested whether parents show the predicted increased reproductive response to reduced survival prospects and this hypothesis is the focus of the present study. To this end, we utilized the experimentally induced variation in the rate of age‐dependent mortality in our study population (Boonekamp et al., 2014, 2015) to test the prediction that reduced somatic state alleviates the restraint on reproductive effort. We first tested whether the effect of brood size treatment on the rate of actuarial senescence holds in a much larger dataset than our initial study to verify this important precondition to test for reproductive restraint. Subsequently, using this extended dataset, we tested for the first time the key prediction that experimentally accelerated age‐dependent mortality rate leads to increased reproductive effort in subsequent years. The bivariate response of survival and reproduction to the experimental treatment allows unravelling of the causal interactions between these two major life‐history variables furthering our understanding of life‐history diversity.

## 2 MATERIALS AND METHODS

### 2.1 Study system

We studied a natural jackdaw *Coloeus monedula* population between 2005 and 2016. Nest boxes were distributed among 11 different breeding sites within a ~2 × ~3 km^2^ area in the vicinity of Groningen (53.1708°N, 6.6064°E, the Netherlands). We visited nest boxes every 3 days to monitor the initiation of nest building and egg laying. Egg width and length were measured to the nearest tenth of a millimetre with which egg volume (*V*) was determined using the formula: $V=\pi \times W^{2}\times L\times K/6$, where *W* is the egg width (mm), *L* is the egg length (mm) and *K* = 0.00096 (Soler, 1988). Once chicks started hatching, we conducted daily nest visits in order to determine the exact hatching dates of nestlings. Hatchlings were weighed and individually marked by clipping the nail tips, which additionally facilitated the collection of small blood droplets (5 µl) to be used for molecular sex determination (for details see Salomons, Dijkstra, & Verhulst, 2008). Nestlings were repeatedly counted, measured and weighed over the nestling period on days 5, 10, 20 and 30 (hatching of the oldest chick = day 1). To enable lifelong identification, nestlings and immigrant adults were ringed with a unique combination of a metal‐numbered ring and colour rings. Identification of ringed birds took place during the nest building and incubation period, that is prior to the manipulation.

### 2.2 Brood size treatment

We aimed to manipulate brood size for the duration of the complete breeding tenure of individuals. To achieve this, we manipulated brood sizes between 2005 and 2016 such that birds received identical treatments each year they returned to breed in the study area. Jackdaws are highly philopatric and breeding birds have a median life span of ~5.5 years and consequently we have manipulated the entire breeding tenures of a large portion of the population (71%). Brood size manipulations were carried out as previously described (Boonekamp et al., 2014): Age‐matched broods were manipulated by adding or removing nestlings at day 5. We manipulated broods such that they contained both resident and cross‐fostered offspring. We transferred three nestlings from reduced to enlarged broods, and one nestling from enlarged to reduced broods, resulting in a net manipulation of two nestlings. When broods contained too few chicks, we moved one nestling from reduced to enlarged broods (6% of cases), and no nestlings were returned. We randomly assigned which nestlings we relocated by using ‘Prime Dice’, a quasi‐randomizer smartphone app. In total, 2,107 nestlings of 544 manipulated broods were included in this study. Natural clutch sizes of parents that received their first manipulation did not differ between reduced and enlarged broods' groups (*M* ± *SD* = 4.75 ± 0.78 vs. 4.55 ± 0.96 respectively). Reduced and enlarged broods contained 1.86 ± 0.88 versus 5.25 ± 1.38 nestlings after the manipulation, and much of this difference persisted until day 30, close to the moment of fledging (reduced: 1.46 ± 0.89 vs. enlarged: 2.91 ± 1.71). The experimental design was optimized to demonstrate an effect of the manipulation on life‐history variables with high statistical power, and consequently does not include a control group with unmanipulated brood sizes. Indeed, our study does not aim to estimate the selection gradient on brood size for which a control group would be necessary.

### 2.3 Survival analyses

We used survival trajectories of 320 individuals between the years 2005–2016 resulting in 908 survival observations. Annual resighting probability of individuals known to be alive was 92%, that is individuals surviving to the next year were not observed to be present in 8% of cases, which is accounted for in the survival estimates (see below). True survival could nevertheless deviate from our survival estimates due to permanent dispersal after breeding for which we cannot control. However, our overall mean survival estimate is similar to the survival estimated by ring recovery data for this species (Dobson, 1990), which is independent of dispersal, suggesting permanent dispersal had little effect on our survival estimates. Moreover, dispersal of breeders between breeding sites happened at a low rate within our study area and did not differ between the two manipulation groups (5%—reduced vs. 4% enlarged). Hence, the survival estimates were unlikely to be biased with respect to the experimental treatment. Jackdaws are highly monogamous, but natural mortality frequently causes the formation of new pairs. Conflicting manipulation trajectories arise when new pairs are formed and when partners differ in their manipulation history. This happened in 6.5% of cases (all other newly formed pairs were with a new recruit to the population), and in such cases the pair was assigned to the manipulation category of the partner with the longest manipulation history and subsequent survival data of the other partner were omitted from the analyses. This right‐censoring of the survival data did not systematically differ among the sexes and/or manipulation groups.

We used Bayesian survival trajectory analysis (BaSTA; Colchero, Jones, & Rebke, 2012, version 1.9.5) to estimate the treatment effect on age‐dependent mortality rate from the onset of the experimental treatment onwards. We have previously shown that this approach is advantageous over classical survival analysis when the purpose is to quantify treatment effects on the shape of the mortality distribution rather than an average survival effect, although results of both approaches were in qualitative agreement (Boonekamp et al., 2014). BaSTA is a useful method in the context of life‐history biology, because the life‐history framework specifically predicts instantaneous mortality probability to increase with age. BaSTA integrates the estimation of mortality and recapture probability parameters. We used a specific set‐up in BaSTA to quantify the effect of the treatment on subsequent mortality trajectories by defining the time of birth as the age of first manipulation in the BaSTA model and by including the age of first manipulation as a covariate to align mortality—age distributions (for details see Boonekamp et al., 2015). BaSTA also enables the fitting and evaluation of a number of differently shaped mortality distributions. The two‐parameter Gompertz distribution was the best supported model compared to an exponential, Weibull or logistic model, and produced a narrow fit (*r* = .88) to the raw mortality data (as estimated by least squares fitting of the Gompertz distribution to the raw life span data). The Gompertz function includes a baseline and an age‐dependent mortality parameter that respectively determine the intercept and slope of a linear mortality–age relationship of the form *u*(*x*) = *b*0 + *b*1 × *x*, where *u*(*x*) is the natural logarithm of mortality rate. We aimed to estimate the effect of the experimental treatment on the two Gompertz parameters, where the effect on *b*1 would represent a difference in actuarial senescence among the two treatment groups. To this end we computed the posterior distributions for the Gompertz parameters for each treatment group. We used the Kullback–Leibler discrepancy calibration (K‐L; Burnham & Anderson, 2001; Kullback & Leibler, 1951) to evaluate the distance among posterior distributions. K‐L values range from 0.5, indicating that two posterior distributions contain identical information, to 1, which indicates completely non‐overlapping information. K‐L values > 0.8 can be considered to indicate a substantial difference in information content among posterior distributions (McCulloch, 1989), indicating a biologically significant difference. Final posterior distributions were based on four individual MCMC simulation chains 110,000 iterations each, 10,000 burn‐in, and 100 sampling interval, resulting in low serial autocorrelations (<10%) and sufficiently large posterior distributions (*n* = 4,000). Note that we have previously confirmed that classical CMR analysis including state‐ and year‐dependent survival supported the results of the BaSTA (Boonekamp et al., 2014).

### 2.4 Reproductive success analyses

We used maximum log likelihood mixed effects models with lme4 (Bates, Mächler, Bolker, & Walker, 2015) to assess the effect of treatment on five reproductive variables in subsequent years: lay date, clutch size, egg volume and nestling survival (note that we made the distinction between pre‐manipulation survival, between egg–day 5, and post‐manipulation survival between days 5 and 30). There can be different ways in which age and treatment affected reproductive success. We therefore took the approach to carefully consider a number of statistical models based on biological plausibility and subsequently applied model selection to evaluate their support by the data. We performed the model selection procedure in two sequential steps: First, the best fitting ‘background’ model was selected, containing only variables that were unrelated to the experimental treatment. Second, building on the best fitting background model, alternative ‘treatment’ models were considered to evaluate the evidence for carry‐over effects of the brood size treatment on reproductive success in subsequent years (Table 1). We evaluated model performance based on the lowest AIC value and selected the best supported models based on ΔAIC values > 2 (Burnham & Anderson, 2002). Below follows a detailed description of the models used in the two sequential model selections.

**Table 1:** (A) Background models (*M*_0_ *X*) considered to identify the best model describing variables that were unrelated to the experimental treatment. We considered the following variables: ‘*t*_first_’ the age at the onset of treatment, ‘*t*_last_’ a factor denoting the last year of reproduction, ‘*t*’ denoting the time (in years) in treatment. We also included two different ways to accommodate annual variation, either by using ‘year’ as a random effect, or by the inclusion of ‘year_mean_’, as a continuous variable reflecting the overall mean per year of the dependent variable of reproductive success that was analysed. Furthermore, we included ‘site’, the location of breeding, and ‘BirdID’, the identity linking reproductive bouts within individuals across years, as random effects, and for the analyses of egg volume we also included ‘NestID’ as a nested random effect within ‘BirdID’. Note that in addition to the mentioned models listed below, background models for nestling survival (days 5 and 30) included the experimental treatment factor ‘exp’, to distinguish the carry‐over effects of brood size treatment to subsequent years from the immediate effects of the treatment on nestling survival. (B) Five models that test the effect of the treatment on reproductive success, building on the best fitting background model ‘*M*_0_ *X*’ in the background model selection. These models included the interaction terms of ‘exp’ × ‘first’ and/or ‘exp’ × ‘*t*’ (or ‘*t* ^2^’) to test for instantaneous carry‐over effects of the experimental treatment ‘exp’ from the first treatment year to subsequent years ‘first’, or to test for gradually accumulating effects with time in treatment ‘*t*’ respectively

(A) Background models
$\left(M_{0}1\right)\;y\;=\;\text{random}\left(\text{year}\;+\;\text{site}\;+\;\text{BirdID}\right)\;+\;\text{fixed}\left(t_{\text{first}}\right)$
$\left(M_{0}2\right)\;y\;=\;\text{random}\left(\text{site}\;+\;\text{BirdID}\right)\;+\;\text{fixed}\left(t_{\text{first}}\;+\;{\text{year}}_{\text{mean}}\right)$
$\left(M_{0}3\right)\;y\;=\;\text{random}\left(\text{year}\;+\;\text{site}\;+\;\text{BirdID}\right)\;+\;\text{fixed}\left(t_{\text{first}}\;+\;t_{\text{last}}\right)$
$\left(M_{0}4\right)\;y\;=\;\text{random}\left(\text{site}\;+\;\text{BirdID}\right)\;+\;\text{fixed}\left(t_{\text{first}}\;+\;{\text{year}}_{\text{mean}}\;+\;t_{\text{last}}\right)$
$\left(M_{0}5\right)\;y\;=\;\text{random}\left(\text{year}\;+\;\text{site}\;+\;\text{BirdID}\right)\;+\;\text{fixed}\left(t_{\text{first}}\;+\;t\right)$
$\left(M_{0}6\right)\;y\;=\;\text{random}\left(\text{site}\;+\;\text{BirdID}\right)\;+\;\text{fixed}\left(t_{\text{first}}\;+\;{\text{year}}_{\text{mean}}\;+\;t\right)$
$\left(M_{0}7\right)\;y\;=\;\text{random}\left(\text{year}\;+\;\text{site}\;+\;\text{BirdID}\right)\;+\;\text{fixed}\left(t_{\text{first}}\;+\;t_{\text{last}}\;+\;t\right)$
$\left(M_{0}8\right)\;y\;=\;\text{random}\left(\text{site}\;+\;\text{BirdID}\right)\;+\;\text{fixed}\left(t_{\text{first}}\;+\;{\text{year}}_{\text{mean}}\;+\;t_{\text{last}}\;+\;t\right)$
$\left(M_{0}9\right)\;y\;=\;\text{random}\left(\text{year}\;+\;\text{site}\;+\;\text{BirdID}\right)\;+\;\text{fixed}\left(t_{\text{first}}\;+\;t\;+\;t^{2}\right)$
$\left(M_{0}10\right)\;y\;=\;\text{random}\left(\text{site}\;+\;\text{BirdID}\right)\;+\;\text{fixed}\left(t_{\text{first}}\;+\;{\text{year}}_{\text{mean}}\;+\;t\;+\;t^{2}\right)$
$\left(M_{0}11\right)\;y\;=\;\text{random}\left(\text{year}\;+\;\text{site}\;+\;\text{BirdID}\right)\;+\;\text{fixed}\left(t_{\text{first}}\;+\;t_{\text{last}}\;+\;t\;+\;t^{2}\right)$
$\left(M_{0}12\right)\;y\;=\;\text{random}\left(\text{site}\;+\;\text{BirdID}\right)\;+\;\text{fixed}\left(t_{\text{first}}\;+\;{\text{year}}_{\text{mean}}\;+\;t_{\text{last}}\;+\;t\;+\;t^{2}\right)$
(B) Selected background model + treatment models
$\left(M1\right)\;y=M_{0}X\;+\;\exp\;+\;\text{first}\;+\;\exp\;\times \;\text{first}$
$\left(M2\right)\;y=M_{0}X\;+\;\exp\;+\;t\;+\;\exp\;\times \;t$
$\left(M3\right)\;y=M_{0}X\;+\;\exp\;+\;\text{first}\;+\;\exp\;\times \;\text{first}\;+\;t\;+\;\exp\;\times \;t$
$\left(M4\right)\;y=M_{0}X\;+\;\exp\;+\;t\;+\;t^{2}\;+\;t^{2}\;\times \;\exp$
$\left(M5\right)\;y=M_{0}X\;+\;\exp\;+\;t\;+\;t^{2}\;+\;t\;\times \;\exp\;+\;t^{2}\;\times \;\exp$

First, we developed background models that differed in how they addressed the annual variation in variables of reproductive success. All background models included female bird ID and breeding site as random effects to account for non‐independence of the data within these grouping factors. We compared models with ‘year’ as a random effect with models that included the annual mean reproductive success as a fixed covariate. The latter resulted in better fits for all of the reproductive success variables that we analysed (Table 1A). In the background model selection, we also evaluated which age pattern was best supported. This is important because our aim was to test the effect of treatment over and above the effects of age. We therefore included the variables ‘*t*_first_’ (the age at which birds received their first brood size manipulation) to control for the among‐individual variation in the age at which they entered the experiment (*M* = 2.0, *SD* = 0.63) and ‘*t*’ (time in treatment), which reflects the longitudinal change in reproductive success within individual females from the onset of treatment onwards. We also tested the quadratic effect of ‘*t* ^2^’ and ‘*t*_last_’, a factor denoting whether individuals were in their last year of reproduction to test for a terminal effect (Table 1A). It is an interesting observation that none of the models that included ‘*t*_last_’ (Models: 3, 4, 7, 8, 11 and 12) were supported in the model selection procedure (Tables 1A and 3), and hence we found no evidence for a sudden change in reproductive success in the last year of reproduction. We assigned immigrant breeders the age of 2, corresponding to the modal age of first reproduction of known local recruits. We also tested the effect of life span as a covariate in our models, but no such effect was detected.

In the second model selection, we extended the best fitting background models with additional terms to test the parental reproductive response, that is the carry‐over effect of the treatment on reproductive success in subsequent years (Table 1B). Thus, we specifically assessed the carry‐over effect of brood size treatment to any future season, rather than being interested in direct effects of the manipulation in the same season. We anticipated that the treatment could affect reproduction trajectories in different non‐mutually exclusive ways with treatment effects arising instantaneously (between the first and subsequent experimental years—then staying stable) and/or gradually (a gradual increase with time in treatment). To this end, we constructed models that tested the interaction between ‘exp’ (a factor denoting whether the brood was reduced (exp = 0) or enlarged (exp = 1) × ‘first’ (a factor denoting whether the brood was the first manipulated brood (first = 0), or a brood in subsequent years (first = 1)) to test for an instantaneous carry‐over effect of the treatment. We included models with the interaction term ‘exp’ × ‘*t*’ (time in treatment) to test for accumulating effects of the longitudinal treatment. Note, we tested for this interaction irrespective of whether the main effect ‘*t*’ was supported in the best background model (as the interaction ‘*t*’ × ‘exp’ may nevertheless be biologically relevant). We also tested models that included both interaction terms to test their additive effects, because instantaneous and accumulating effects are not mutually exclusive. Finally, we included the interaction of ‘exp’ × ‘*t* ^2^’ to test for nonlinear patterns with time in treatment (Table 1B). With respect to the treatment effect we only consider the carry‐over effect to subsequent years over and above any direct effect of brood size manipulation on reproductive success. This means that the main effect of treatment had to be included in the background model for post‐manipulation nesting survival (between day 5 and 30).

## 3 RESULTS

### 3.1 Age‐dependent mortality

**FIGURE 2:**
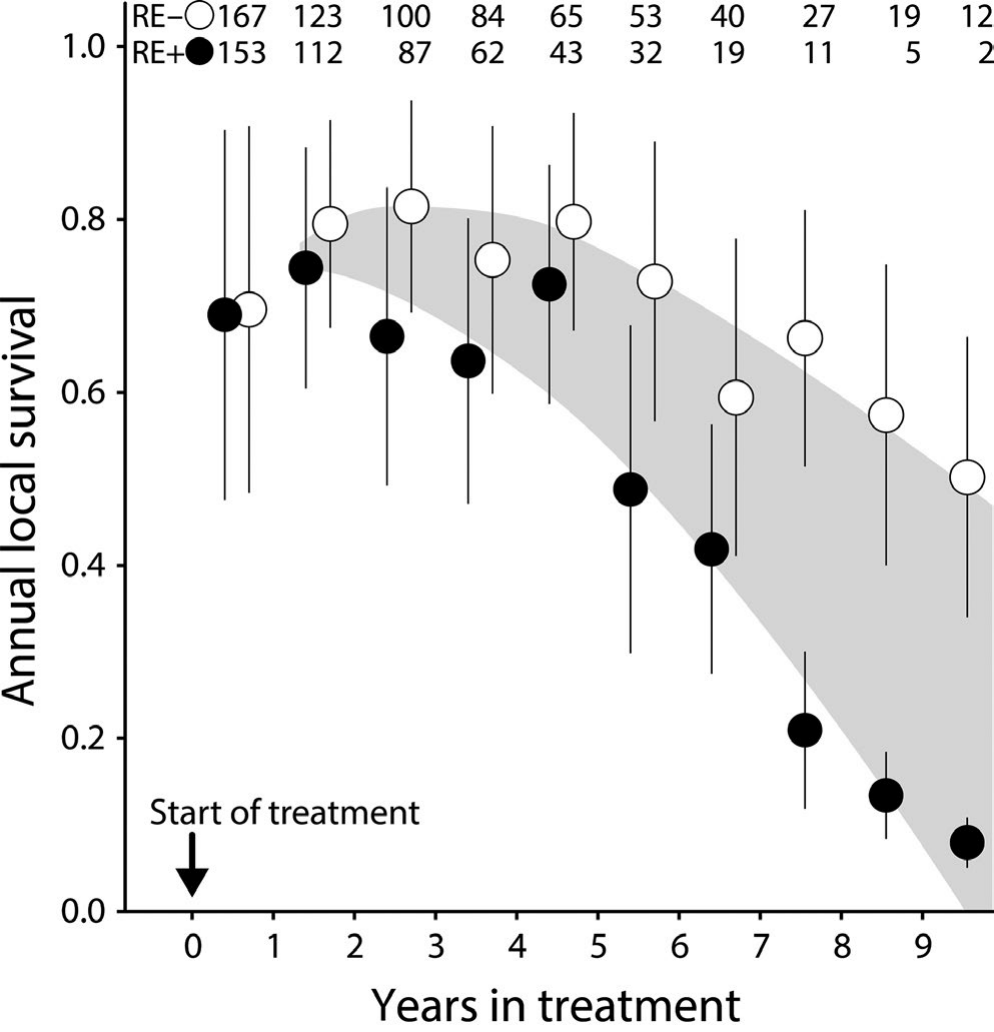
Annual local survival probability (i.e. corrected for the average recapture probability) in relation to longitudinal brood size treatment in a wild jackdaw population. In the top of the figure are the sample sizes of adults in the reduced (RE−) versus the increased effort (RE+) treatment groups. The vertical bars represent the standard error bars based on a binomial error distribution. Birds received their first manipulation in year 0 and survived on average 69% from year 0 to year 1. The shaded area, drawn by eye, serves to illustrate how the average survival difference between treatment groups evolved over time

**FIGURE 3:**
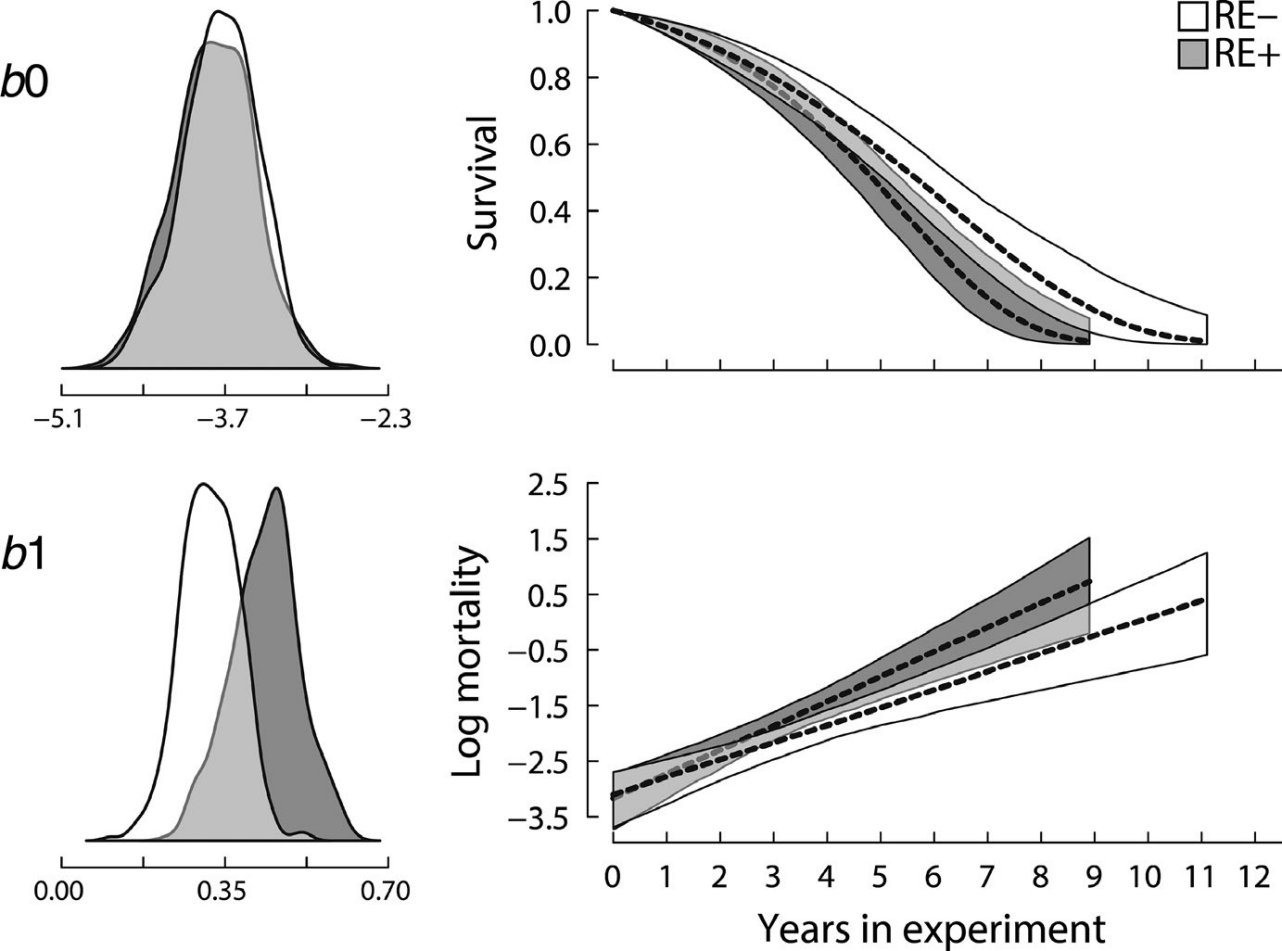
Cumulative survival probability (top right panel) and the natural log of instantaneous mortality rate (bottom right panel) in relation to time in treatment (in years) in a wild jackdaw population. RE− and RE+ indicate the reduced and increased reproductive effort treatment groups respectively. Panels on the left show posterior distributions of Gompertz parameters ‘*b*0’ (baseline mortality) and ‘*b*1’ (age‐dependent mortality). Note that the mean of the posterior ‘*b*0’ and ‘*b*1’ reflect the intercept and the slope of the mortality trajectories in the lower panel on the right. Shaded areas reflect the 95% confidence intervals inferred from the posterior distributions

Since our earlier report (Boonekamp et al., 2014, 2015), sample size has increased from 186 to 320 individuals, and from 469 to 987 bird‐years. We therefore repeated the survival analysis to verify whether our initial conclusion that rearing enlarged broods accelerates actuarial senescence holds for the expanded dataset we use here to test for reproductive restraint. Mean annual adult survival probability was 69% in the first year of manipulation and was indistinguishable between the two treatment groups (Figure 2); a result that mirrors the results of a meta‐analysis of single‐year manipulation studies (Santos & Nakagawa, 2012). The raw survival data show that the survival trajectories start to diverge between the two treatment groups after the initial treatment year, with survival declining more rapidly in the RE+ group (Figure 2). To evaluate the statistical support for the apparent diverging survival trajectories between treatments, we fitted a two‐parameter Gompertz mortality distribution to the survival data using a Bayesian approach (see Section 2 for details). We found that *b*1 (the slope of a linear relationship between years in treatment and the natural logarithm of instantaneous mortality rate) was 1.56‐fold higher in the birds rearing enlarged broods (RE+) relative to the birds rearing reduced broods (RE−), while baseline mortality rate *b*0 (the intercept) was unaffected by treatment (Table 2; Figure 3). The difference in mortality trajectory resulted in a 24% higher remaining life expectancy from the onset of treatment in the RE− treatment (4.7 years), relative to the RE+ treatment (3.8 years) group (Table 2). There was a weak evidence that average female life expectancy was 0.5 years longer than that of males, and that the experimental effect on *b*1 was larger in females, but neither difference was statistically convincing (Table 2). The estimates of *b*0 and *b*1 overlap with our earlier estimates (Boonekamp et al., 2014, 2015) and both analyses show that the manipulation effect arises via an effect on *b*1 with indistinguishable *b*0 among treatment groups. Considering that the present analysis includes many birds, this replication of our initial finding increases confidence in the treatment effect. The finding that rearing an increased brood size for life considerably accelerated age‐dependent mortality rate provides both empirical support for a critical assumption of life‐history theory as well as ensuring the appropriate conditions for a test of the reproductive restraint hypothesis (Figure 1).

**Table 2:** Bayesian survival trajectory analysis estimating the effect of lifelong brood size manipulation on the mortality trajectories of 320 breeding jackdaws. Mortality trajectories were fitted using the two‐parameter Gompertz equation, which describes a linear relationship between years in manipulation and the natural logarithm of instantaneous mortality rate. We used the DIC (divergence information criterion—a Bayesian alternative to AIC) to formally compare the information performance of the null model versus the alternative model that included the brood size manipulation grouping factor. The DIC of the alternative model was 19 lower indicating that brood size manipulation affected the pattern of actuarial senescence substantially. We subsequently used the Kullback–Leibler discrepancy calibration (Burnham & Anderson, 2001) (K‐L) to quantify the information distance between the posterior distributions of *b*0 and *b*1 among the two treatment groups (see Section 2 for details). K‐L values close to 0.5 indicate completely overlapping posterior distributions and K‐L values > 0.8 indicate a substantial information distance (McCulloch, 1989). The effect of the manipulation on *b*1, but not *b*0, resulted in an 24% difference in life expectancy (L.E.) from the onset of treatment. C.P. denotes the annual probability to observe or recapture a surviving individual and this value is used in BaSTA to estimate survival

	Manipulation	DIC	*b*0 (*SE*)	K‐L (*b*0)	*b*1 (*SE*)	K‐L (*b*1)	L.E.	C.P.
No covariates	NA	1688	−2.28 (0.21)	NA	0.18 (0.04)	NA	–	0.92
Brood size treatment	Reduced	1669	−2.37 (0.26)	0.50	0.16 (0.05)	0.91	4.7	0.92
	Enlarged		−2.40 (0.27)		0.25 (0.06)		3.8	
Treatment × sex	♀ reduced	1772	−2.24 (0.27)	0.50	0.12 (0.06)	0.81	5.0	0.92
	♀ enlarged		−2.30 (0.32)		0.21 (0.09)		4.3	
	♂ reduced		−2.43 (0.29)		0.22 (0.07)	0.71	4.5	
	♂ enlarged		−2.34 (0.31)		0.29 (0.08)		3.7	

### 3.2 Reproductive success

We applied a two‐step model selection approach to test for a reproductive response to the experimental treatment using lay date, clutch size, egg volume, pre‐ and post‐manipulation nestling survival (pre: from egg to day 5 and post: from day 5 to fledging) as variables of reproductive success. In the first step, the best model was selected to control for variables unrelated to the experimental treatment (background models; Table 1A). Building on the selected background model the second model selection step then commenced, adding treatment‐related variables to test for carry‐over effects of the treatment on reproductive success in subsequent years (treatment models; Table 1B).

Lay date, clutch size and pre‐manipulation offspring survival in subsequent years were not affected by the treatment, as these variables were best supported by the background model without additional variables related to the experiment (Table 3). In contrast, model selection revealed that treatment affected egg volume and post‐manipulation nestling survival (Table 3). These effects were caused by carry‐over effects of the experimental treatment in previous years on these traits in subsequent years, increasing in parents rearing enlarged broods relative to parents rearing reduced broods.

**Table 3:** Sequential two‐step model selection based on AIC for the reproductive variables lay date, egg volume, clutch size, pre‐manipulation nestling survival (egg–day 5) and post‐manipulation nestling survival (day 5 and 30). The table header ‘Model’ denotes models comparing background (*M*_0_) and treatment (*M*) models as described in Section 2 and Table 1, and ‘delta AIC’ gives the difference in the AIC values relative to the best fitting model. Hence, the best fitting background models have a delta AIC value of zero. These selected background models were subsequently used in the treatment model selection (i.e. the bottom section of the table). Numbers in brackets in the treatment model selection indicate AIC differences of the best fitting treatment model relative to the best fitting background model. Shadings indicate the lowest AIC values of the two sequential model selections combined and include the *R* ^2^ value showing the model fit. Egg volume and nestling survival (day 5–30) were best described by the *M*1 and *M*5 treatment models, respectively, indicating significant carry‐over effects of the treatment on reproductive success in subsequent years

Model	delta AIC				
	Lay date	Egg volume	Clutch size	Survival (egg‐day 5)	Survival (day 5−30)
Background model selection					
*M*_0_1	40	32	7	3	18
*M*_0_2	0|0.62	22	0|0.26	0|0.20	5
*M*_0_3	42	33	9	4	20
*M*_0_4	2	24	2	2	7
*M*_0_5	40	29	8	5	16
*M*_0_6	1	0	2	1	7
*M*_0_7	41	28	10	6	19
*M*_0_8	2	1	3	2	9
*M*_0_9	41	29	8	7	10
*M*_0_10	2	1	2	1	0
*M*_0_11	42	30	10	7	12
*M*_0_12	3	1	4	2	2
Treatment model selection					
*M*1	2	0 (−32)|0.63	2	0 (+0)	5
*M*2	0 (+3)	4	0 (+1)	1	10
*M*3	2	2	4	4	6
*M*4	3	9	2	2	11
*M*5	3	6	4	2	0 (−9)|0.61

**FIGURE 4:**
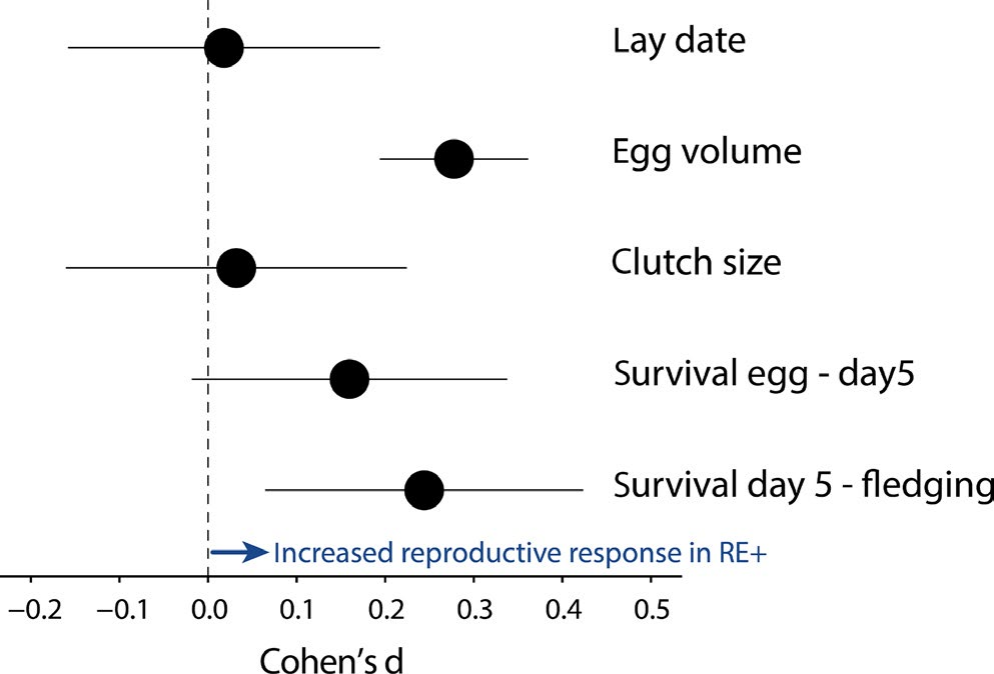
Mean reproductive response to the brood size treatment of five variables of reproductive success and their 95% confidence intervals. Effect sizes are shown in Cohen's *d* values, which estimate the mean difference in reproductive success (i.e. from the first to subsequent experimental years) among the two treatment groups. Positive values indicate that reproductive success increased in RE+ (increased effort) relative to the RE− (reduced effort) experimental groups. Cohen's *d* values were calculated based on the *t* value of the ‘first × exp’ interaction term using the *M*1 model (Table 1)

The results from the model selection procedure were supported by post hoc testing of the interaction between treatment group and the factor ‘first’, denoting whether the manipulated brood was in the first or in subsequent years of the lifelong experiment. This interaction term estimates the among‐treatments difference in the mean reproductive response from the first to subsequent experimental years. Based on this interaction term we calculated the Cohen's *d* effect size, which was close to zero for lay date and clutch size, showed a positive trend for pre‐manipulation nestling survival and was significantly positive for egg volume and post‐manipulation nestling survival probability to the fledging age (Figure 4).

**FIGURE 5:**
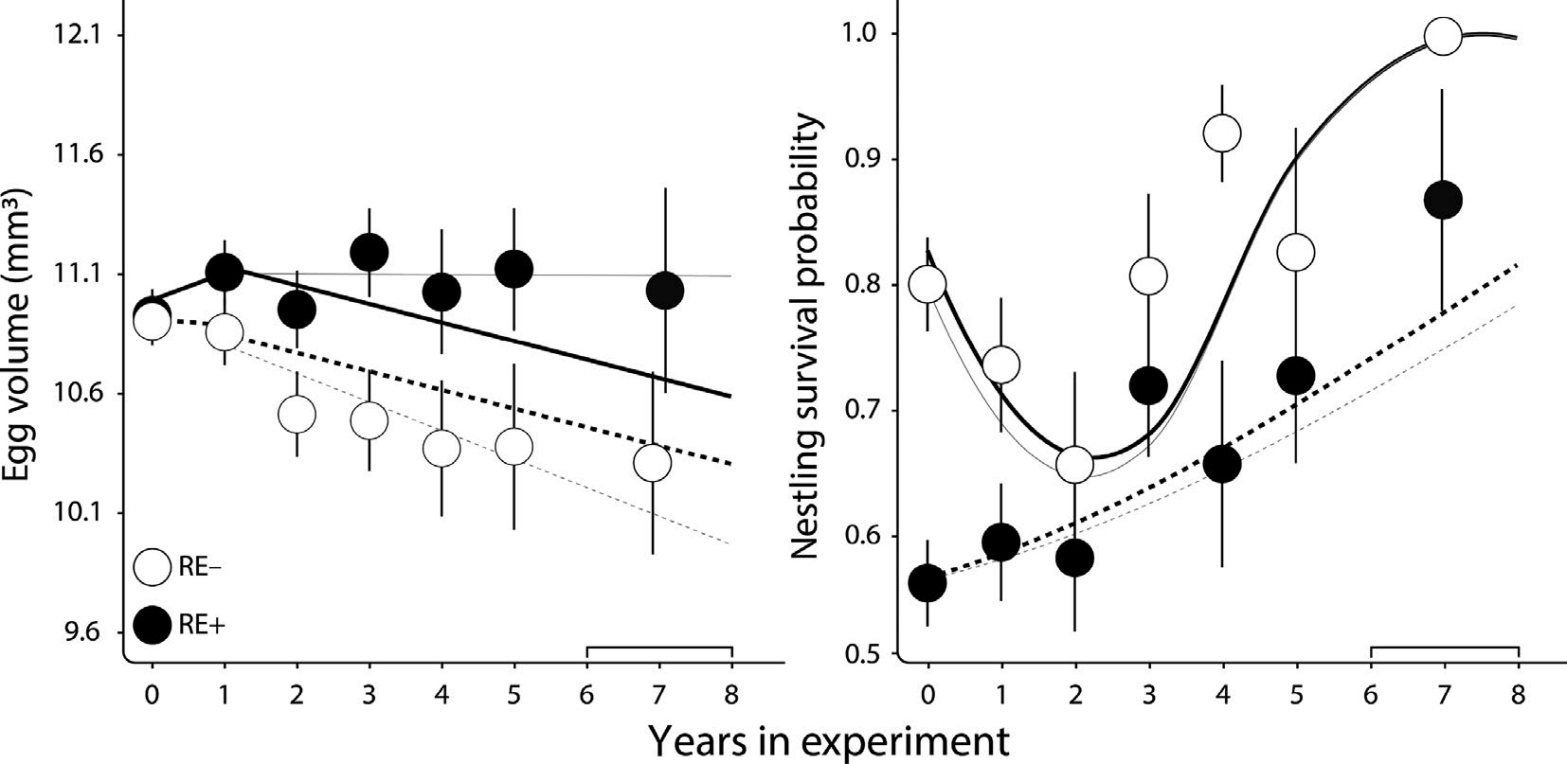
Egg volume (mm^3^ ± *SE*) and nestling survival probability between days 5 and 30 (±*SE*) in relation to the experimental treatment. RE− (dotted line) and RE+ (solid line) indicate reduced and increased reproductive effort brood size treatment groups respectively. Year 0 denotes the first year of treatment. Years 6–7 were pooled due to limited sample sizes in these groups. Regression lines in bold reflect the best supported longitudinal models shown in Table 4. Thin regression lines show the fit of a cross‐sectional model (see Section 3 for details). Note that nestling survival in enlarged broods was lower on average than in reduced broods due to a strong negative direct effect of the manipulation. This direct effect however does not capture the carry‐over effect (the reproductive response from one year to the next) that was the focus of this study

Egg volume, a variable known to have long‐term fitness consequences in our study system (Verhulst & Salomons, 2004), decreased on average with time in treatment (Table 4; Figure 5) reflecting a within individual decline of egg volume with age. The best supported model for egg volume (*M*1) did not include the interaction between time in treatment × treatment group, but the second‐best model (*M*3) did include this interaction and with very similar support as M1 (Table 3). Figure 5 suggests that there could be a gradual effect of treatment on egg volume, increasing with time in treatment. However, post hoc testing revealed that the interaction of time in treatment × treatment was not significant in the *M*3 model. Furthermore, even though the interaction was significant in the *M*2 model (*p* = .028), the AIC value of model *M*2 was 4 higher relative to the *M*1 model (Table 3), indicating weak support for a gradual effect of treatment on egg volume. Upon closer inspection of Figure 5 it can be seen that the regression lines of model *M*1 do not seem to accurately overlay the egg volume data points, despite that these lines represent the model with the strongest support from our model selection. One possible explanation for the apparent discrepancy is that our models are longitudinal, in the sense that they estimate the within individual change in egg volume as a function of time in treatment, but the data points in Figure 5 are cross sectional, showing the pattern with time in treatment of the combined within (time in treatment) and among (selection) individual processes. We therefore ran the cross‐sectional model including the interaction of time in treatment × treatment group (i.e. *M*2 without the random effect BirdID) to analyse the cross‐sectional pattern with time in treatment, and visual inspection shows regression lines estimated from this model to fit much better to the data (Figure 5). The difference between the longitudinal and cross‐sectional models suggests that selective disappearance confounds inference from the cross‐sectional pattern, causing the longitudinal regression lines to deviate from the cross‐sectional data as shown in Figure 5. To explore the potential extent of selective disappearance in more detail we divided the time in treatment variable into an average and delta time component, similar to the established average age versus delta age approach (van de Pol & Wright, 2009). We then tested whether the average time component, reflecting the among‐individual variation of time in treatment, differed between the two treatment groups. Indeed, the interaction of treatment group × average time in treatment was significant (0.27 ± 0.12, *p* = .027), indicating that the slopes of average time differed between the two manipulation groups: in the RE+ group the average time in treatment slope was positive (0.08 ± 0.12), whereas in the RE− group the slope was negative (−0.194 ± 0.08). These results suggest that the relationship between egg volume and survival are different between the two treatments, with egg volume being positively associated with survival in the RE+ group. However, because the average time in treatment slope did not significantly differ from zero, the correlation between egg volume and survival in the RE+ group does not appear to be significant.

**Table 4:** Parameter estimates of the best supported treatment models of egg volume and nestling survival, being the two variables where model selection showed there to be treatment effects. ‘*n*’ denotes sample sizes of the number of eggs, nests, birds and breeding sites; ‘*σ* ^2^ random’ reflect variance components of random effects (nest, bird and breeding site) and residuals (note that residual variance is 1 in logistic regression used to analyse nestling survival). The ‘fixed effects’ reflect the fixed effects that were included in the model and their estimates, standard errors and *p*‐values. The fixed effects were: ‘year_mean_’ the mean annual reproductive success, ‘*t*_first_’ the age at first treatment, ‘*t*’ time in treatment, ‘exp’ the experimental brood size treatment group (0 = reduced; 1 = enlarged), and ‘first’ a factor denoting whether the brood was in the first (first = 0) or subsequent (first = 1) year of experimental treatment. The survival model contained four fewer broods due to early nestling mortality

Variable	*n*	*σ* ^2^ random		Fixed effects	Estimate (*SE*)	*p*
Egg volume	2252_egg_/490_nest_/187_bird_/11_site_	nestID	0.065	year_mean_	0.895 (0.095)	<.001
		birdID	0.595	*t*_first_	0.127 (0.090)	.161
		site	0.002	*t*	−0.078 (0.017)	<.001
		residual	0.417	exp	0.025 (0.025)	.844
				first	0.027 (0.027)	.702
				first × exp	0.214 (0.088)	.015
Survival day 5 and 30	486_nest_/187_bird_/11_site_	birdID	0.57	year_mean_	0.063 (0.006)	<.001
		site	0.37	*t*_first_	0.135 (0.139)	.332
		residual	NA	*t*	−1.019 (0.241)	<.001
				exp	−1.470 (0.252)	<.001
				*t* ^2^	0.218 (0.055)	<.001
				*t* × exp	0.951 (0.272)	<.001
				*t* ^2^ × exp	−0.199 (0.062)	.001

For nestling survival, the best supported model (*M*5) showed a gradual increase in log survival with time in treatment in the RE+ group, whereas there was a quadratic relationship between time in treatment and nestling survival in the RE− group, showing a decline in the first 3 years of treatment after which it increased (Table 4; Figure 5). Post hoc testing supported both the decline observed in the first three treatment years in the RE− group (*p* = .002), and the increase thereafter (*p* = .006), as well as the gradual increase observed in the RE+ group (*p* = .040). We also compared the fit of a cross‐sectional model (i.e. without BirdID), which produced a very similar fit (Figure 5).

## 4 DISCUSSION

**FIGURE 6:**
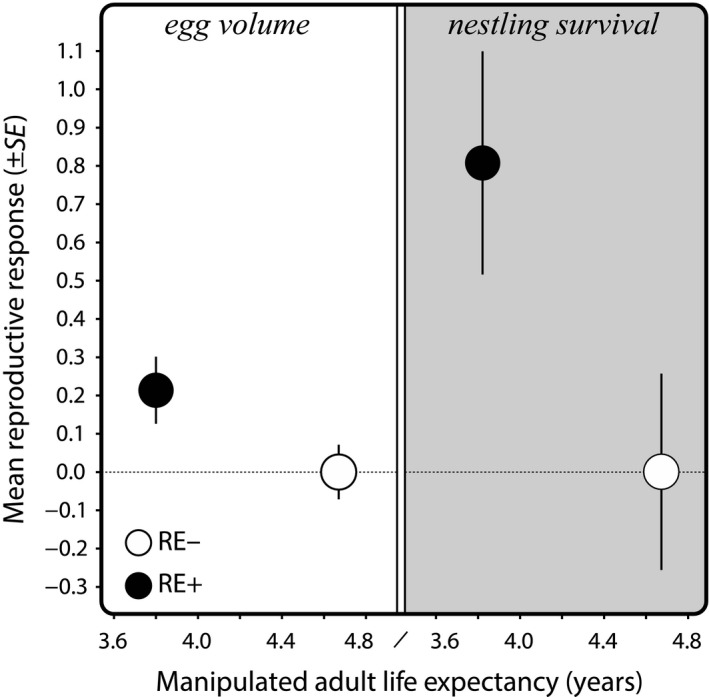
Mean reproductive response (egg volume mm^3^ and nestling survival probability between day 5 and 30 ±*SE*) in relation to manipulated adult life expectancy. Values reflect relative responses to the RE− group (set to 0 as reference). Plotted values were derived from the interaction term of the *M*1 models for egg volume and nestling survival (see Table 1), and hence show the average effect over life after the first treatment year independent of age. Life expectancy values were derived from the life tables as estimated by BaSTA (Table 2)

An evincible cost of reproductive effort on the residual life span is an important precondition to demonstrate reproductive restraint. To our best knowledge, our finding that experimentally increased reproductive effort induced an increased reproductive response in subsequent years, where this is shown simultaneously with confirmation that this manipulation reduced life span (Figure 6) is the first such demonstration in a vertebrate. Our findings are supported by a study showing reproductive restraint in *Cladocerans* in the laboratory through the manipulation of perceived predation risk, which enhanced early reproduction and shortened life span (Dawidowicz, Prędki, & Pietrzak, 2010; Pietrzak, Dawidowicz, Prędki, & Dańko, 2015). Several other studies that manipulated the perception of survival prospects, albeit without measuring survival, yielded very similar results. For example, administration of an immune challenge consistently resulted in higher reproductive success (Bonneaud, Mazuc, Chastel, Westerdahl, & Sorci, 2004; Bowers et al., 2012; Cotter, Ward, & Kilner, 2011; Sköld‐Chiriac, Nilsson, & Hasselquist, 2018; Velando, Drummond, & Torres, 2006). In a similar vein, reduced barometric air pressure, simulating approaching bad weather, increased reproductive effort in a parasitic wasp in the laboratory (Roitberg, Sircom, Roitberg, Alphen, & Mangel, 1993) and another study showed that host immune effectors accelerated growth and reproduction of filarial parasites (Babayan, Read, Lawrence, Bain, & Allen, 2010). However, when an experimental effect on survival probability is not demonstrated it remains speculative whether the treatment modulated somatic state, and the possibility remains that the observed reproductive responses were caused by some other mechanism unrelated to perceived remaining life span. Our study provides that additional confirmation in a natural population. These and our findings together suggest that reproductive restraint is likely to be widespread in nature, occurring both in vertebrates and invertebrates. Reproductive restraint is therefore likely to be an important factor underpinning natural variation in age‐specific reproductive success and may help explaining the commonly observed increase in reproductive success from the early to mid‐life stages (Curio, 1983; Martin, 1995).

Previous studies of reproductive responses following brood size manipulation in birds show heterogeneous results (Dijkstra et al., 1990; Parejo & Danchin, 2006) and similar variation has been observed in wild mammals (e.g. Clutton‐Brock, 1984; Gélin, Wilson, Coulson, & Bianchet, 2015; Hamel et al., 2011) and a wild insect population (Rodríguez‐Muñoz et al., 2019). The observed heterogeneity may have multiple explanations. Firstly, for either intrinsic (e.g. energy turnover) or extrinsic (e.g. time or food limitation) reasons, there may be a constraint on a further increase in reproductive effort (Tinbergen & Verhulst, 2000). In this scenario animals do not have the option to show an increase in reproductive effort. Secondly, the experimentally induced increase in reproductive effort is likely to have a carry‐over effect on the physiological and/or cognitive state of the animals, as evidenced by the acceleration of actuarial senescence that we observed in our present study. State may affect reproductive efficiency, modulating by how much reproductive effort influences reproductive success. For example, state may have an effect on foraging efficiency (Limmer & Becker, 2009). The observed reproductive success may be the outcome of two opposing effects; an increase in reproductive effort and a decline in state. That we observed an increase in reproductive success suggests that the increase in reproductive effort outweighed a potential negative effect of reduced somatic state in our experiment, but there are also examples where reproductive success was reduced in the year following an experimentally induced increase in reproductive effort (Gustafsson & Pärt, 1990; Verhulst, 1998) suggesting the balance went the other way. Thus, although an observed increase in reproductive success agrees with life‐history theory, deviations of this pattern do not necessarily prove it wrong.

Williams predicted that if a single physiological mechanism, such as damage accumulation in somatic cells, is responsible for senescence, then different performance traits should senesce more or less in concert (Williams, 1957). According to this idea, we would also expect our manipulation to affect different reproductive traits similarly, but instead we found remarkable variation in reproductive responses to the experimental reduction in remaining life expectancy among the different reproductive traits (Figure 4). In egg volume, there was an instantaneous effect from the first treatment to subsequent years and the treatment effect persisted and remained stable, while laying date and clutch size were unaffected. For nestling survival, in contrast, there was a quadratic effect of time in treatment and the shape of these curves differed between treatments with a remarkable decrease in nestling survival of the RE− group over the first 3 years of treatment, followed by a sharp increase in nestling survival from 4 years in treatment onwards (Figure 5). We found no terminal effects for reproduction traits, but previously reported terminal declines in telomere length (Salomons et al., 2009) and social dominance (Verhulst, Geerdink, Salomons, & Boonekamp, 2014) in this population. The heterogeneity in treatment effect is reminiscent of heterogeneity in age‐specific patterns of reproductive success among reproductive traits in vertebrates (Bouwhuis & Vedder, 2017; Hayward et al., 2015) and also in wild insects (Rodríguez‐Muñoz et al., 2019), and perhaps has the same explanation. This heterogeneity could reflect variation among performance traits in their net contribution to fitness (Boonekamp, Mulder, & Verhulst, 2018; Gaillard & Lemaitre, 2017). Parents may prioritize allocation to reproductive traits that yield the highest net fitness returns at the expense of traits that contribute less strongly to fitness, a hypothesis that we have previously confirmed for non‐reproductive variables in the same study system (Boonekamp, Dijkstra, Dijkstra, & Verhulst, 2017; Boonekamp, Mulder, et al., 2018). Differential resource allocation may underpin the observed asynchrony in age‐trajectories among performance traits and explain the heterogeneity of responses in our present study, but this hypothesis remains to be tested.

The inverse relationship we observed between remaining life expectancy and reproductive effort (Figure 6) raises the question what physiological mechanism(s) underlies the modulation of resource allocation. Telomere length and rate of attrition have attracted considerable attention, because of their association with survival in a range of species in the wild (Wilbourn et al., 2018), including jackdaws (Salomons et al., 2009). There is also increasing evidence that telomere dynamics are related reproductive success (Sudyka, 2019) and hence, telomere dynamics could mechanistically underpin the relationship between survival and reproductive restraint. However, in our wild jackdaw study system we found that the lifelong brood size manipulation that affected adult age‐dependent mortality had no noticeable effect on the rate of adult telomere attrition (Bauch et al., in prep.). This suggests that telomere dynamics are unlikely to causally underpin the observed trade‐off between reproduction and survival in our study system. Resolving this mechanism remains an important question for the future, for practical reasons and because it may shed light on the enigma of ageing.

## AUTHORS' CONTRIBUTIONS

S.V. conceived the study; All authors contributed to the field work; Brood size manipulations were planned and carried out by J.J.B.; Data analyses were done by J.J.B. and S.V.; J.J.B. wrote the first draft of the manuscript. All authors subsequently contributed to the final version of the paper.

## Data Availability

Data available from the Dryad Digital Repository: https://doi.org/10.5061/dryad.8cz8w9gks (Boonekamp, Bauch, & Verhulst, 2020).
